# Genetic basis analysis of key Loci in 23 Yannong series wheat cultivars/lines

**DOI:** 10.3389/fpls.2022.1037027

**Published:** 2022-10-10

**Authors:** Luning Xiao, Yuli Jin, Wei Liu, Jie Liu, Huajie Song, Dong Li, Jianpeng Zheng, Dongmei Wang, Yan Yin, Yang Liu, Hao Wang, Linzhi Li, Nina Sun, Minxiao Liu, Pengtao Ma

**Affiliations:** ^1^ College of Life Sciences, Yantai University, Yantai, China; ^2^ Institute of Grain and Oil Crops, Yantai Academy of Agricultural Sciences, Yantai, China; ^3^ Rongcheng Science and Technology Bureau, Rongcheng, China; ^4^ Shandong Seed Administration Station, Jinan, China

**Keywords:** markers, PHS, *Pm* genes, *Yr* genes, drought resistance

## Abstract

Fungal diseases, drought, pre-harvest sprouting (PHS) and other biotic and abiotic stresses have seriously affected the quality and yield in wheat production. Identifying related genes/loci in released cultivars/lines can provide reference information and theoretical basis for wheat improvement. Yannong series wheat cultivars/lines have distinctive characteristics in wheat cultivars and play an important role in genetic improvement and production of Chinese wheat production system. To dissect their genetic basis of the stress-resistant traits, in this study, 23 representative Yannong series wheat cultivars/lines were tested by 58 molecular markers for 40 genes related to adaptability, disease resistance and stress tolerance to clarify the genetic composition of the key loci. The results showed that most of the tested wheat accessions carried dwarfing genes *RhtB1b*/*RhtD1b*/*Rht8* and recessive vernalization genes *vrn-A1*/*vrn-B1*/*vrn-D1*/*vrn-B3*. It was also consistent with the phenotypic traits of tested Yannong series wheat which were dwarf and winter or semi winter wheat. In addition, the overall level of seedling powdery mildew resistance in 23 Yannong wheat cultivars/lines was moderate or inadequate. Eleven accessions carried none of the tested *Pm* genes and twelve accessions carried *Pm2*, *Pm6*, *Pm42* and *Pm52* singly or in combination. Then, 23 wheat cultivars/lines were also tested by 17 diagnostic markers for 14 *Yr* genes. The results showed that 16 wheat cultivars/lines were likely to carry one or more of tested *Yr* genes, whereas Yannong 15, Yannong 17, Yannong 23, Yannong 24, Yannong 377, Yannong 572 and Yannong 999 carried none of the tested *Yr* genes. Moreover, in our study, nine markers for four genes related to drought tolerance and PHS were used to evaluate the stress tolerance of the 23 wheat cultivars/lines. The results indicated that all 23 wheat cultivars/lines carried drought resistance genes *Ta-Dreb1/TaCRT-D*, indicating that they had the drought resistance to the extent. Except for Yannong 30, Yannong 377, Yannong 390, Yannong 745 and Yannong 1766, other wheat cultivars/lines carried one to three elite PHS-resistant alleles *Vp-1Bc/Vp-1Bf/TaAFP-1Bb*.

## Introduction

Bread wheat (*Triticum aestivum* L.) is one of the food crops widely planted in the world and related to food security and social stability. However, its yield and quality are constantly challenged by various biotic and abiotic stresses (Ma et al., 2014; Zhang Y. W. et al., 2017). Systematically evaluating the adaptability, disease resistance and stress tolerance of released wheat cultivars/lines at molecular and genetic level is momentous to rationally distribute and apply them.

Powdery mildew caused by the biotrophic fungus *Blumeria graminis* f. sp. *tritici* (*Bgt*) and stripe (yellow) rust created by *Puccinia striiformis* f. sp. *tritici* (*Pst*) are the most widespread and damaging fungal diseases of wheat, which limit wheat harvest in many parts of the world (Kokhmetova et al., 2014; Zhang et al., 2016). To date, more than 80 formally named *Pm* genes (Li et al., 2020; McIntosh et al., 2020; He et al., 2021) and 80 *Yr* genes (McIntosh et al., 2020) have been identified, respectively. The great majority of these genes are race-specific, dominant and easy to lose resistance independently used. Recently, powdery mildew and stripe rust has become more severe and epidemic with changing of cultivation measures and environment, such as changes in irrigation, rising of nitrogen fertilizer and the climate change (Tang et al., 2017). Therefore, wheat cultivars/lines are easier to become susceptible under the high disease pressure and more rapid evolution of virulent pathogen population. Identifying resistance genes in released wheat cultivars/lines is essential to determine which resistance genes becoming ineffective and utilize the effective and known resistance genes for the reasonable arrangement of disease-resistance cultivars and genes.

In addition to biotic diseases, drought and pre-harvest sprouting (PHS) are two common abiotic stresses during the growth and development of wheat. PHS, refers to germination process of physiologically ripe seeds in the ear, ie, before harvest, happens in almost each wheat growing areas in the world (Vetch et al., 2019). It results in the production of protein and starch degradative enzymes that degrade endosperm protein and starch to supply energy for germination (Ross and Bettge, 2009). There are lots of physiological factors related to PHS. Among them, the principal are dormancy and ear morphology. Dormancy which is a complicated quantitative trait has serious effects on PHS susceptibility (Rodriguez et al., 2015). The transcription factor *VP1*, is crucial to regulate wheat dormancy and PHS. The specific sequence-tagged site (STS) marker *Vp1B3* for gene *VP1* can amplify three types of bands. Among them, the bands of 849 bp and 569 bp are PHS resistant, whereas 652 bp is PHS susceptible (Yang et al., 2007; Chang et al., 2010). In the same way, nearly half of the whole land for wheat growth is influenced by periodic drought (Pfeiffer et al., 2004). It is estimated that a 10% reduction in wheat production will occur because of drought or extreme weather conditions by statistical models (Lesk et al., 2016). The severity of effect is determined by species genotype, developmental phase, organ type and length of stress period. The responses of plant to drought stress are miscellaneous due to the kinds of processes occurring at macro and micro levels (Kiani et al., 2020). To solve the problem of abiotic stress, the breeding goals and strategies should change from conventional breeding to advanced molecular design breeding.

Molecular markers assisted selection breeding is deemed as a useful, quick and easy way to identify known genes/loci present in released wheat materials. The functional markers of known genes that are generally developed based on polymorphic sites within the genes and directly related to phenotypic variations are the ideal breeding markers (Liu et al., 2012; Jin et al., 2021). In wheat, many genes could be detected by corresponding functional markers, for example, *Rht-B1b*, *Rht-D1b* (Ellis et al., 2002), *Yr15* (Peng et al., 2000), *Pm2* (Jin et al., 2021), *Pm21* (Bie et al., 2015), *Pm24* (Lu et al., 2020) and *Vp1-B* (Yang et al., 2007; Chang et al., 2010). However, lots of genes have not been cloned yet, and their detection depends on the closely linked or co-segregated markers. Among kinds of molecular markers, simple sequence repeat (SSR), STS markers and single-nucleotide polymorphism (SNP) markers play a key role in studying genes associated with multifarious traits (Keller et al., 1999; Liu et al., 2002; Wu et al., 2019).

Yannong series wheat cultivars/lines, developed by Shandong Yantai Academy of Agricultural Sciences (Yantai, China), have distinctive characteristics and play an important role in Chinese wheat production system. Numerous wheat cultivars have been derived from Yannong series cultivars, of which 284, 210, 77, 30, 23 and 17 cultivars have been derived from Youbaomai (Wang et al., 2021), Lumai 14 (Sun et al., 2020a), Lumai 13 (Sun et al., 2020b), Yannong 19 (Yin et al., 2019), Lumai 21 (Sun et al., 2019a) and Yannong 15 (Sun et al., 2019b), respectively. The promotion areas of Yannong series wheat cultivars have reached 39.69 million hm^2^ in production (Liu et al., 2019). In our study, to identify the genetic basis and make better use of Yannong series cultivars, we intended to evaluate their characteristics, disease-resistance and stress tolerance using molecular markers, which will be useful for utilization of Yannong series cultivars in production.

## Materials and methods

### Plant materials

The 23 wheat cultivars/lines were developed and provided by Shandong Yantai Academy of Agricultural Sciences (**Table S1**). The pedigrees of these accessions are listed in **Table S1**. Wheat landrace cultivar Huixianhong (HXH) was susceptible to powdery mildew and used as the susceptible check in resistance evaluation experiment. Fifteen wheat lines carrying known *Pm* genes and fourteen lines carrying known *Yr* genes were also used as positive control in molecular marker detection experiment (**Table S2**). The donors of *Yr* genes were provided by Prof. Caixia Lan, College of Plant Science and Technology, Huazhong Agricultural University.

### Agronomic performance

The 13 representative cultivars/lines (**Table 1**) were planted at Yantai National Crop Variety Regional Test Station (37° 65′ 59″ N, 120° 47′ 01″ E) from 2021 to 2022 in a randomized complete block design with three replicates. Each cultivar/line was planted as a plot with four rows (1.5 m length and 0.25 m between rows) and 30 seeds per row. Randomly ten wheat plants in the middle of the second and third rows were selected to evaluate the spike numbers per plant (SNPP), plant height (PH), spike length (SL), spikelet numbers per spike (SNS), kernel numbers per spike (KNS), thousand-kernel weight (TKW) and kernel related traits. SNPP and PH were assessed according to the mean of ten plants. SNS, SL and KNS were identified depended on the mean of main spike of ten plants. TKW was determined after gathering in the crops through weighing three samples of 500 kernels.

**Table 1:** Agronomic traits evaluation of 13 Yannong cultivars/lines.

Cultivars/lines	PH (cm)	SL (cm)	SNPP	SNS	KNS	TKW (g)
Yannong 30	71.4 ± 1.9	9.0 ± 0.3	12.0 ± 1.0	15.3 ± 1.3	54.3 ± 2.3	55.0 ± 0.0
Yannong 37	76.6 ± 2.0	8.3 ± 0.6	8.7 ± 0.3	19.7 ± 2.3	53.0 ± 1.0	53.4 ± 0.1
Yannong 377	77.4 ± 4.5	8.9 ± 0.1	8.3 ± 0.3	18.0 ± 3.0	59.7 ± 2.3	51.2 ± 0.0
Yannong 15	85.4 ± 2.9	7.8 ± 0.1	9.7 ± 4.3	19.7 ± 0.3	44.3 ± 2.3	33.5 ± 0.0
Yannong 17	75.1 ± 2.8	6.6 ± 0.3	9.0 ± 1.0	16.7 ± 1.3	59.3 ± 0.3	47.3 ± 0.0
Yannong 31	77.1 ± 0.1	9.3 ± 0.1	9.3 ± 1.3	18.7 ± 1.3	53.7 ± 1.3	54.2 ± 0.0
Yannong 215	74.7 ± 3.1	7.1 ± 0.7	5.7 ± 0.3	18.7 ± 2.3	54.0 ± 4.0	53.5 ± 0.3
Yannong 572	66.5 ± 0.5	7.9 ± 0.1	9.0 ± 1.0	18.0 ± 1.0	47.3 ± 0.3	51.2 ± 0.1
Yannong 745	74.6 ± 4.4	7.6 ± 0.3	7.7 ± 0.3	16.3 ± 2.3	45.3 ± 2.3	52.4 ± 0.1
Yannong 999	82.7 ± 1.3	6.8 ± 2.8	8.7 ± 2.3	16.7 ± 2.3	45.7 ± 4.3	56.4 ± 0.1
Yannong 1212	77.8 ± 2.2	7.0 ± 0.2	9.0 ± 1.0	20.3 ± 4.3	51.0 ± 3.0	51.6 ± 0.1
Lumai 14	85.2 ± 0.9	9.5 ± 0.2	8.7 ± 0.3	18.7 ± 0.3	42.7 ± 4.3	47.2 ± 0.0
Lumai 21	81.4 ± 3.1	8.4 ± 0.4	12.7 ± 4.3	18.3 ± 0.3	54.3 ± 2.3	36.1 ± 0.0

PH, plant height; SL, spike length; SNPP, spike numbers per plant; SNS, spikelet numbers per spike; KNS, kernel numbers per spike; TKW, thousand-kernel weight.

### Resistance assessment to powdery mildew

Twenty-three cultivars/lines were tested with nine different *Bgt* isolates, including A3, A10, A45, E09, E15, E18, E20, E21 and E32, in the greenhouse at College of Life Science, Yantai University with a condition of 14 h/light/22°C and 10 h/darkness/18°C. Ten seeds of each cultivar/line were sown in rectangular trays with 128 cells and the size of each cell was 3.2 × 3.2 × 4.2 cm, and the susceptible check HXH was planted in three cells in every tray randomly. All seedlings were inoculated with fresh powdery mildew spores at the stage of one leaf. Seedlings in different rectangular trays were inoculated with the nine powdery mildew *Bgt* isolates, separately, then covered a glass shroud on each tray to avoid cross contamination of different isolates after inoculation. When the pustules were fully developed on the first leaf of susceptible control HXH about 14-15 days after inoculation, infection types (ITs) for each plant were assessed on a 0-4 scale, and plants with ITs 0-2 were regarded as resistant and those with ITs 3 and 4 susceptible (Si et al., 1987).

### Molecular marker analysis

Total genomic DNA was isolated using the cetyltrimethylammonium bromide (CTAB) method from wheat young leaf tissues (Sharp et al., 1988). To determine the presence of the excellent genes in these 23 wheat cultivars/lines, 60 diagnostic markers related to wheat adaptability, disease-resistance and stress tolerance were used to test them, including three markers for three dwarfing genes, nine markers for four vernalization genes, 20 markers for 15 *Pm* genes and 17 markers for 14 *Yr* genes, six markers for two drought tolerance genes, three markers for two PHS tolerance genes. The information of molecular markers was described in **Table S3**.

A 10 μl volume was used for polymerase chain reactions (PCR) amplification, containing 5 μl 2 × *Taq* Master Mix (Vazyme, China), 1 μl 50 ng/μl template DNA and 0.5 μl 10 μM/μl primers. The PCR amplification conditions were as follows: pre-denaturation at 94°C for 5 min, 36 cycles of 94°C for 30 s, 50 to 65°C (based on the different primers) for 40 s, 72°C for 40 s to 120 s (according to the size of target bands), finally extension at 72°C for 10 min and preservation at 25°C. PCR products were separated in either 8% non-denaturing polyacrylamide gels with 29:1 ratios of acrylamide and bis-acrylamide, then silver stained and visualized as previously described (Santos et al., 1993), or 1.5% agarose gel, then visualized using the Gel Documentation System (Gel Doc XR+, BIO-RAD, Hercules, CA, USA) (Gebrewahid et al., 2020).

Kompetitive Allele Specific PCR (KASP) markers *YTU-KASP-Pm2* (Yu et al., 2022) was performed in a 8 μl volume which consisted of 4 μl 2 × KASP Master Mix (LGC Company, UK), 2.4 μl 50 ng/μl template DNA, 0.15 μl primer mix (12 μM HEX primer and FAM primer, respectively, 30 μM reverse primer) and 1.45 μl ddH_2_O. PCR procedure was carried out with a Bio-Rad CFX Connect real-time PCR system (BIO-RAD, USA): 95°C for 15 min, followed by ten touchdown cycles (95°C for 20 s; touchdown at 64°C initially and decreasing by -0.6°C per cycle for 60 s), 38 regular cycles of 94°C for 20 s and 58°C for 60 s. Finally, fluorescence was detected and analyzed at 20°C using Bio-Rad CFX Manage 3.1 software.

### Homologous cloning of powdery mildew resistance gene *Pm2* in wheat cultivars/lines

Since wheat cultivars Yannong 31 and Yannong 337 carried powdery mildew resistance gene *Pm2* based on the results of molecular marker detection, the homologous genomic sequences of *Pm2* in these two cultivars were cloned using the primer Pm2b-F/R (F: 5’-3’ ATGGCTGCCTCTGCTGCACTCG, R: 5’-3’ GTGCAACGACGACTCGGACATA), then, compared with the cloned *Pm2* sequence (Sánchez-Martín et al., 2016).

## Results

### Evaluation of agronomic performance

**Figure 1:**
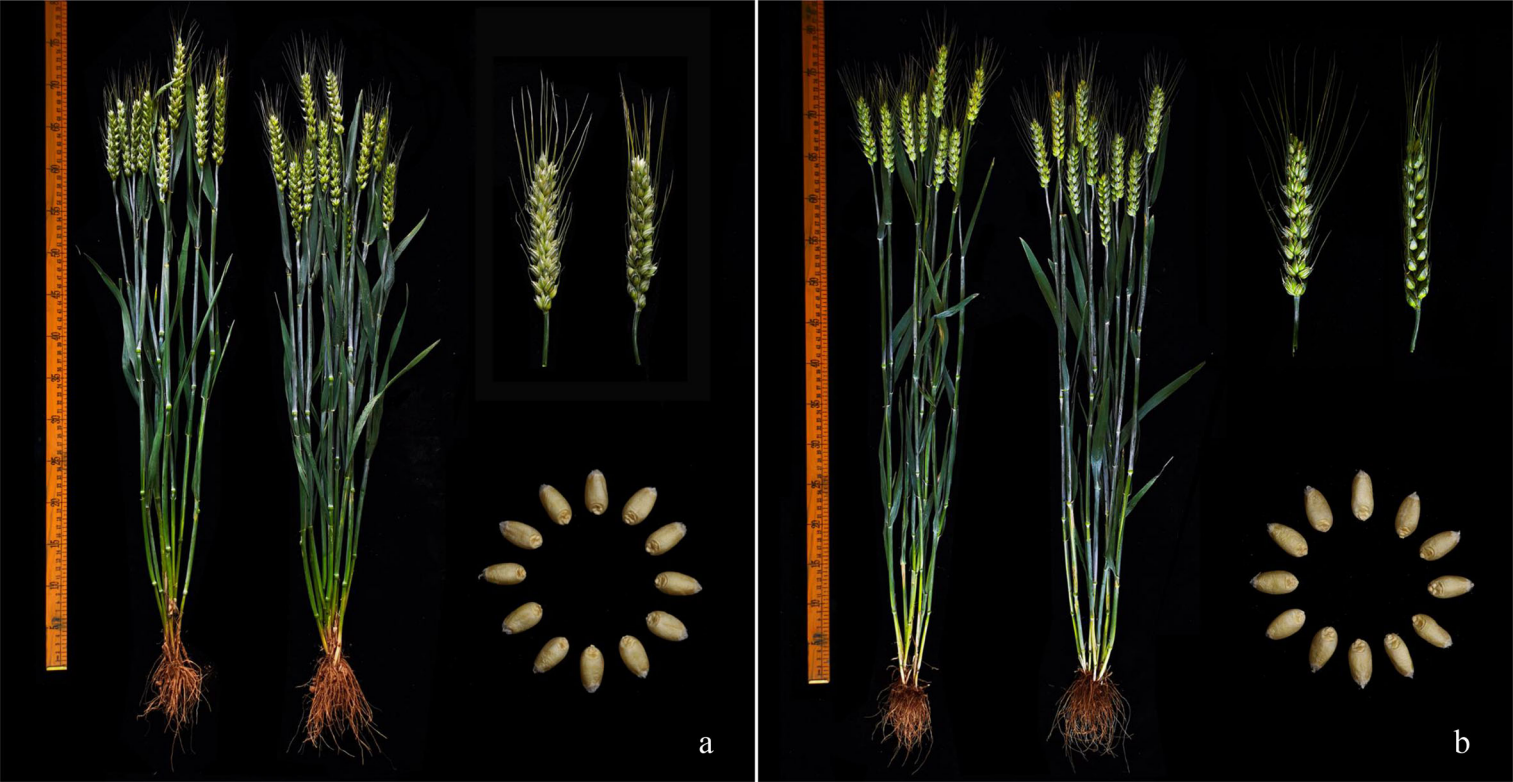
Morphological traits of wheat cultivar Yannong 31 **(A)** and Yannong 1212 **(B)**.

To make better use of the Yannong series wheat cultivars/lines in breeding programs, from 2021 to 2022, 13 wheat cultivars/lines were selected to assess their comprehensive agronomic traits. All these cultivars/lines showed comprehensively excellent performance for the investigated traits, including PH, SL, SNPP, SNS, KNS and TKW (**Table 1**, **Figure 1**), and no any disadvantages were detected. This is coincident with the generally elite production performance of these culitivars in different regions.

### Identification of dwarfing genes and vernalization genes

**Figure 2:**
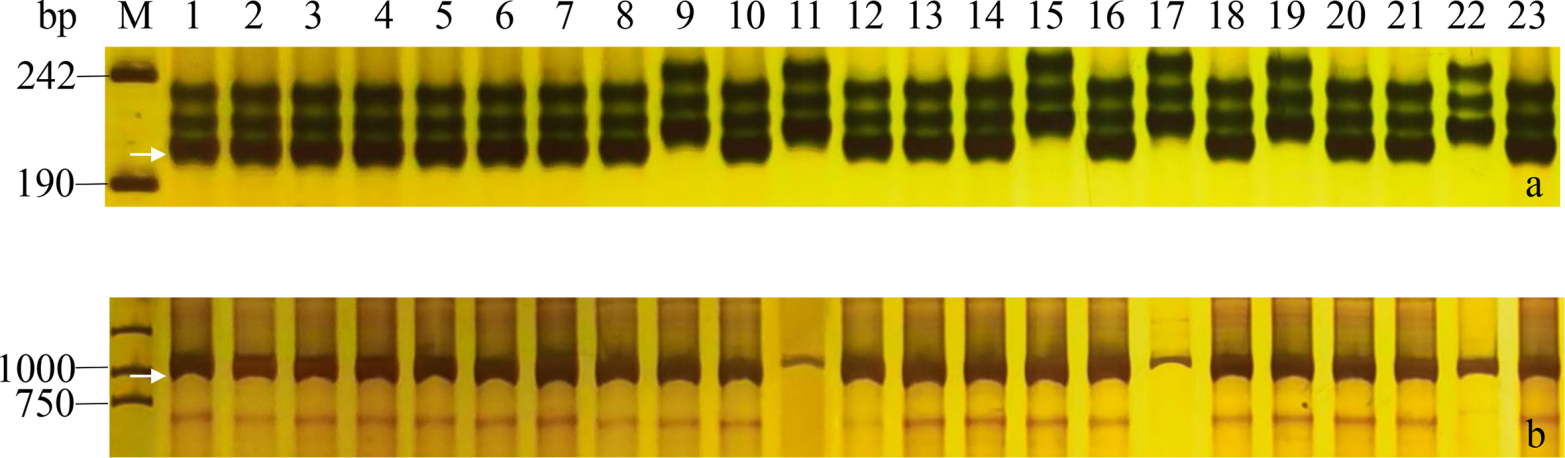
PCR amplifications of specific markers *GWM261* for *Rht8* **(A)** and *Intr1/D* for *vrn-D1* **(B)** in 23 Yannong series wheat cultivars/lines. M, pUC19 **(A)**/DL2000 **(B)**; Lane 1-23: Yannong 30, Yannong 37, Yannong 377, Yannong 15, Yannong 17, Yannong 23, Yannong 24, Yannong 31, Yannong 161, Yannong 191, Yannong 215, Yannong 301, Yannong 390, Yannong 572, Yannong 745, Yannong 836, Yannong 999, Yannong 1212, Yan 2415, Yannong 5158, Lumai 21, Lumai 14, Yannong 1766. The white arrows indicate the specific amplified fragments.

In the present study, the markers for three dwarfing genes *RhtB1b*, *RhtD1b* and *Rht8* were used to investigate the 23 wheat cultivars/lines. The STS marker *BF-MR1* for *RhtB1b* and *DF-MR2* for *RhtD1b* amplified 237 bp and 254 bp target bands in all tested accessions, respectively. The SSR marker *GWM261* for *Rht8* amplified the specific fragment about 192 bp in 17 wheat cultivars/lines except for Lumai 14, Yannong 161, Yannong 215, Yannong 745, Yannong 999 and Yan 2415. The results indicated that these 23 Yannong series cultivars/lines carried at least two dwarfing genes (**Table 2**, **Figure 2**). Additionally, the genetic diversity of vernalization genes determines the broad adaptability of wheat. We have also detected four vernalization genes *Vrn-A1/vrn-A1*, *Vrn-B1/vrn-B1*, *Vrn-D1/vrn-D1* and *Vrn-B3/vrn-B3* using nine diagnostic markers. Overexpression of these dominant alleles could accelerate flowering and maturity of wheat (Yan et al., 2003). In this study, 21 of 23 wheat cultivars/lines carried recessive genes *vrn-A1*, *vrn-B1*, *vrn-D1* and *vrn-B3.* Yannong 215 and Yangnong 999 carried recessive genes *vrn-A1*, *vrn-B1*, *vrn-B3* and dominant gene *Vrn-D1* (**Table 2**, **Figure 2**).

**Table 2:** Detection of dwarfing genes and vernalization genes with molecular markers in 23 Yannong cultivars/lines.

Cultivars/lines	Dwarfing gene			Vernalization gene							
	*Rht-B1b*	*Rht-D1b*	*Rht8*	*Vrn-A1c*	*vrn-A1*	*Vrn-B1*	*vrn-B1*	*Vrn-D1*	*vrn-D1*	*Vrn-B3*	*vrn-B3*
Yannong 15	+	+	+	–	+	–	+	–	+	–	+
Yannong 17	+	+	+	–	+	–	+	–	+	–	+
Yannong 23	+	+	+	–	+	–	+	–	+	–	+
Yannong 24	+	+	+	–	+	–	+	–	+	–	+
Yannong 30	+	+	+	–	+	–	+	–	+	–	+
Yannong 31	+	+	+	–	+	–	+	–	+	–	+
Yannong 37	+	+	+	–	+	–	+	–	+	–	+
Yannong 161	+	+	–	–	+	–	+	–	+	–	+
Yannong 191	+	+	+	–	+	–	+	–	+	–	+
Yannong 215	+	+	–	–	+	–	+	+	–	–	+
Yannong 301	+	+	+	–	+	–	+	–	+	–	+
Yannong 377	+	+	+	–	+	–	+	–	+	–	+
Yannong 390	+	+	+	–	+	–	+	–	+	–	+
Yannong 572	+	+	+	–	+	–	+	–	+	–	+
Yannong 745	+	+	–	–	+	–	+	–	+	–	+
Yannong 836	+	+	+	–	+	–	+	–	+	–	+
Yannong 999	+	+	–	–	+	–	+	+	–	–	+
Yannong 1212	+	+	+	–	+	–	+	–	+	–	+
Yan 2415	+	+	–	–	+	–	+	–	+	–	+
Yannong 5158	+	+	+	–	+	–	+	–	+	–	+
Lumai 21	+	+	+	–	+	–	+	–	+	–	+
Lumai 14	+	+	–	–	+	–	+	–	+	–	+
Yannong 1766	+	+	+	–	+	–	+	–	+	–	+

+, gene present; –, gene absent.

### Evaluation of powdery mildew resistance and identification of known *Pm* genes

**Figure 3:**
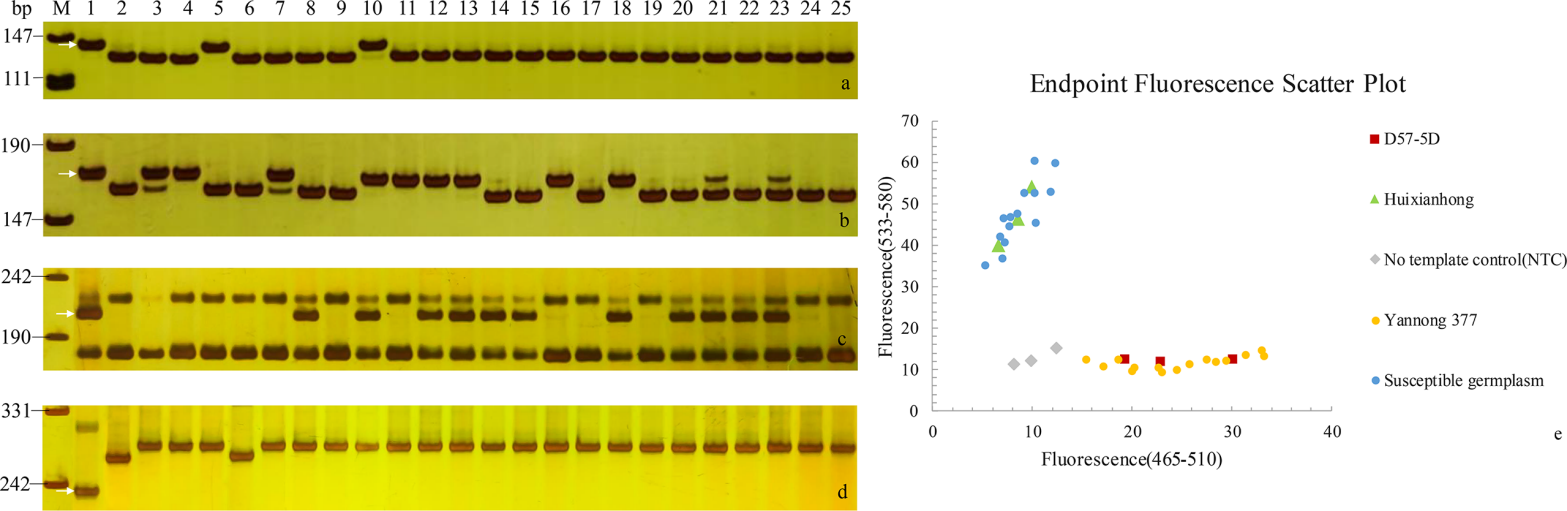
PCR amplifications of specific markers *Pm2b-map-3* for *Pm2* **(A)**, *Xicssl795* for *Pm52* **(B)**, *SC200* for *Yr10* **(C)** and *Y15K1-F2/UHW301R* for *Yr15* **(D)** in 23 Yannong series wheat cultivars/lines. M, pUC19; 1: Positive control; 2: Negative control; 3-25: Yannong 30, Yannong 37, Yannong 377, Yannong 15, Yannong 17, Yannong 23, Yannong 24, Yannong 31, Yannong 161, Yannong 191, Yannong 215, Yannong 301, Yannong 390, Yannong 572, Yannong 745, Yannong 836, Yannong 999, Yannong 1212, Yan 2415, Yannong 5158, Lumai 21, Lumai 14, Yannong 1766. The white arrows indicate the specific amplified fragments. **(E)**, the genotyping results of *YTU-KASP-Pm2* in 23 Yannong series wheat cultivars/lines.

The powdery mildew ITs of 23 wheat cultivars/lines to all nine tested *Bgt* isolates were listed in **Table 3**. Six cultivars/lines, including Yannong 161, Yannong 836, Yannong 1212, Yannong 5158, Yannong 1766 and Lumai 14, were susceptible to all tested isolates at the seedling stage, accounting for 26.09% of tested accessions. Yannong 31, Yannong 191, Yannong 15 and Yannong 37 were highly resistant with IT 0 to four, four, three and three of tested nine *Bgt* isolates at the seedling stage, respectively. The remaining 13 accessions were resistant to only one or two tested isolates. To further detect the presence of known *Pm* genes, 20 diagnostic molecular markers for 15 known *Pm* genes, including *Pm1*, *Pm2*, *Pm4a*, *Pm5e*, *Pm6*, *Pm12*, *Pm21*, *Pm24*, *Pm33*, *Pm34*, *Pm35*, *Pm42*, *Pm45*, *Pm47* and *Pm52*, were selected to test these 23 wheat cultivars/lines. Based on the results, these 23 wheat cultivars/lines could be divided into three groups. The first group showed that Yannong 31 might carry *Pm2+6+52*, Yannong 17 might carry *Pm42+52*, and Yannong 30 and Yannong 191 might carry *Pm6+52*. The second group included eight cultivars/lines which might carry just one of the tested *Pm* genes, referring to one, two and five wheat cultivars/lines that were amplified successfully with diagnostic markers for *Pm2*, *Pm6* and *Pm52*, respectively. No target PCR products were produced by the whole of the tested markers in eleven accessions of the third group, indicating that these cultivars/lines carried none of these tested *Pm* genes. In addition, none of 23 wheat cultivars/lines carry *Pm1*, *Pm4*, *Pm5*, *Pm12*, *Pm21*, *Pm24*, *Pm33*, *Pm34*, *Pm35*, *Pm45* and *Pm47* (**Table 3**, **Figure 3**).

**Table 3:** Seedling infection types to *Blumeria graminis* f. sp. *tritici* and resistance genes detected with molecular markers.

Cultivars/lines	*Blumeria graminis* f. sp. *tritici* (*Bgt*) isolates^a^									Powdery mildew (*Pm*) resistance genes^b^														
	A3	A10	A45	E09	E15	E18	E20	E21	E32	*1*	*2*	*4a*	*5e*	*6*	*12*	*21*	*24*	*33*	*34*	*35*	*42*	*45*	*47*	*52*
Huixianhong	4	4	4	4	4	4	4	4	4	–	–	–	–	–	–	–	–	–	–	–	–	–	–	–
Yannong 15	4	3	3	4	4	0	4	0	0	–	–	–	–	–	–	–	–	–	–	–	–	–	–	–
Yannong 17	0	4	4	4	4	4	4	3	4	–	–	–	–	–	–	–	–	–	–	–	+	–	–	+
Yannong 23	0	4	4	4	4	4	0	3	4	–	–	–	–	–	–	–	–	–	–	–	–	–	–	–
Yannong 24	4	4	4	4	4	4	0	4	3	–	–	–	–	–	–	–	–	–	–	–	–	–	–	–
Yannong 30	0	3	4	0;	4	4	4	4	3	–	–	–	–	+	–	–	–	–	–	–	–	–	–	+
Yannong 31	3	3	3	4	0	3	0	0	0	–	+	–	–	+	–	–	–	–	–	–	–	–	–	+
Yannong 37	0	3	4	0;	4	0	4	4	4	–	–	–	–	–	–	–	–	–	–	–	–	–	–	+
Yannong 161	4	4	4	4	4	4	4	4	4	–	–	–	–	–	–	–	–	–	–	–	–	–	–	+
Yannong 191	4	4	4	4	0	0	0	0	4	–	–	–	–	+	–	–	–	–	–	–	–	–	–	+
Yannong 215	3	4	4	4	3	0	4	0	4	–	–	–	–	–	–	–	–	–	–	–	–	–	–	+
Yannong 301	3	4	0	4	4	0	4	4	4	–	–	–	–	–	–	–	–	–	–	–	–	–	–	–
Yannong 377	4	4	4	0;	4	0	4	4	3	–	+	–	–	–	–	–	–	–	–	–	–	–	–	–
Yannong 390	0	3	4	4	4	4	4	3	0	–	–	–	–	+	–	–	–	–	–	–	–	–	–	–
Yannong 572	4	4	4	4	4	4	4	0	4	–	–	–	–	–	–	–	–	–	–	–	–	–	–	+
Yannong 745	4	3	3	4	4	0	4	0	4	–	–	–	–	–	–	–	–	–	–	–	–	–	–	–
Yannong 836	4	4	3	4	4	4	4	4	4	–	–	–	–	–	–	–	–	–	–	–	–	–	–	+
Yannong 999	4	4	3	4	4	4	4	0	0	–	–	–	–	–	–	–	–	–	–	–	–	–	–	–
Yannong 1212	4	3	4	4	4	4	4	4	4	–	–	–	–	+	–	–	–	–	–	–	–	–	–	–
Yan 2415	0	4	4	4	4	0	3	4	3	–	–	–	–	–	–	–	–	–	–	–	–	–	–	–
Yannong 5158	4	3	4	4	4	4	4	4	3	–	–	–	–	–	–	–	–	–	–	–	–	–	–	–
Lumai 21	0	4	4	4	4	4	3	4	4	–	–	–	–	–	–	–	–	–	–	–	–	–	–	–
Lumai 14	4	3	4	4	4	4	4	3	4	–	–	–	–	–	–	–	–	–	–	–	–	–	–	–
Yannong 1766	4	4	4	4	4	4	3	4	4	–	–	–	–	–	–	–	–	–	–	–	–	–	–	–

a Infection types: 0 = no visible symptoms; 0; = hypersensitive necrotic flecks; 3 = colonies with well-developed hyphae and abundant conidia, but colonies not joined together; and 4 = colonies with hyphae and abundant conidia, and colonies mostly joined together.

b +, gene present; –, gene absent.

Notably, in this study, the cloned powdery mildew resistance gene *Pm2* was detected in wheat cultivars Yannong 31 and Yannong 377 with the functional marker *Pm2b-map-3* and KASP marker *YTU-KASP-Pm2.* To further determine whether there were sequence variation of *Pm2* between these two cultivars and donor of *Pm2*, the genomic sequences of *Pm2* in wheat cultivars Yannong 31 and Yannong 377 were cloned. The results showed that their sequences were completely consistent with cloned *Pm2* sequence (Sánchez-Martín et al., 2016).

### Identification of known *Yr* genes

To investigate the known *Yr* genes, 16 diagnostic molecular markers for 14 known *Yr* genes, including *Yr1*, *Yr5*, *Yr9*, *Yr10*, *Yr15*, *Yr17*, *Yr18*, *Yr24*, *Yr26*, *Yr29*, *Yr30*, *Yr41*, *Yr67* and *YrSP*, were selected to test these 23 wheat cultivars/lines (**Table S3**; **Table 4**). The results showed that 16 wheat cultivars/lines were likely to carry one or more of tested *Yr* genes, whereas Yannong 15, Yannong 17, Yannong 23, Yannong 24, Yannong 377, Yannong 572 and Yannong 999 carried none of the tested *Yr* genes. The target band of *Yr10* was detected in 10 of 23 wheat cultivars/lines with the highest frequency 37%. The marker *H20* for *Yr9* amplified the specific target bands in nine wheat accessions. The markers *Xgwm155* for *YrSP* and *SC2372* for *Yr17* amplified the polymorphic bands in two and one wheat accessions, respectively, suggesting that these cultivars/lines might carry the corresponding *Yr* genes (**Table 4**, **Figure 3**).

**Table 4:** Detection of stripe rust (*Yr*) resistance genes in 23 Yannong cultivars/lines.

Cultivar/line	Stripe rust (*Yr*) resistance genes													
	*Yr1*	*Yr5*	*Yr9*	*Yr10*	*Yr15*	*Yr17*	*Yr18*	*Yr24*	*Yr26*	*Yr29*	*Yr30*	*Yr41*	*Yr67*	*YrSP*
Yannong 15	–	–	–	–	–	–	–	–	–	–	–	–	–	–
Yannong 17	–	–	–	–	–	–	–	–	–	–	–	–	–	–
Yannong 23	–	–	–	–	–	–	–	–	–	–	–	–	–	–
Yannong 24	–	–	–	–	–	–	–	–	–	–	–	–	–	–
Yannong 30	–	–	+	–	–	–	–	–	–	–	–	–	–	–
Yannong 31	–	–	–	+	–	–	–	–	–	–	–	–	–	–
Yannong 37	–	–	+	–	–	–	–	–	–	–	–	–	–	–
Yannong 161	–	–	+	–	–	–	–	–	–	–	–	–	–	–
Yannong 191	–	–	+	+	–	–	–	–	–	–	–	–	–	–
Yannong 215	–	–	–	+	–	–	–	–	–	–	–	–	–	–
Yannong 301	–	–	–	+	–	–	–	–	–	–	–	–	–	–
Yannong 377	–	–	–	–	–	–	–	–	–	–	–	–	–	–
Yannong 390	–	–	–	+	–	–	–	–	–	–	–	–	–	+
Yannong 572	–	–	–	–	–	–	–	–	–	–	–	–	–	–
Yannong 745	–	–	+	–	–	–	–	–	–	–	–	–	–	–
Yannong 836	–	–	+	+	–	–	–	–	–	–	–	–	–	–
Yannong 999	–	–	–	–	–	–	–	–	–	–	–	–	–	–
Yannong 1212	–	–	+	+	–	–	–	–	–	–	–	–	–	+
Yan 2415	–	–	–	+	–	–	–	–	–	–	–	–	–	–
Yannong 5158	–	–	–	+	–	–	–	–	–	–	–	–	–	–
Lumai 21	–	–	–	+	–	–	–	–	–	–	–	–	–	–
Lumai 14	–	–	+	–	–	–	–	–	–	–	–	–	–	–
Yannong 1766	–	–	+	–	–	+	–	–	–	–	–	–	–	–

+, gene present; –, gene absent.

### Identification of genes associated with stress tolerance

**Figure 4:**
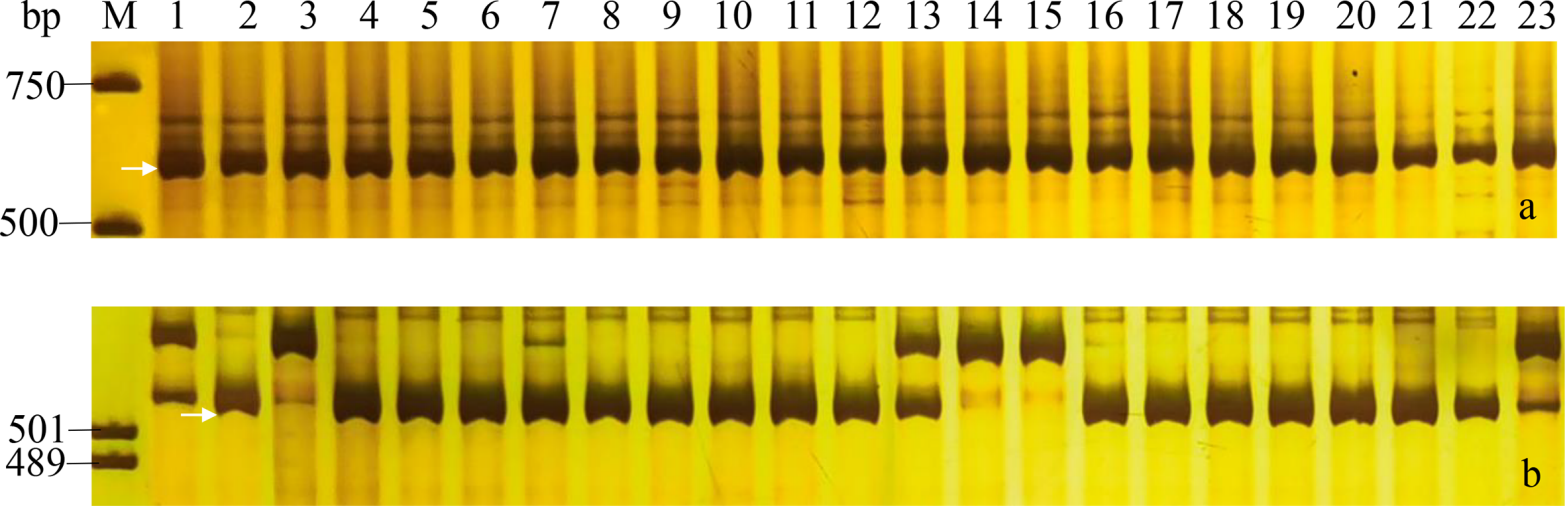
PCR amplifications of specific markers *P22* for *Dreb1* **(A)** and *Vp1B3* for *Vp-1B* **(B)** in 23 Yannong series wheat cultivars/lines. M, DL2000 **(A)**/pUC19 **(B)**; Lane 1-23: Yannong 30, Yannong 37, Yannong 377, Yannong 15, Yannong 17, Yannong 23, Yannong 24, Yannong 31, Yannong 161, Yannong 191, Yannong 215, Yannong 301, Yannong 390, Yannong 572, Yannong 745, Yannong 836, Yannong 999, Yannong 1212, Yan 2415, Yannong 5158, Lumai 21, Lumai 14, Yannong 1766. The white arrows indicate the specific amplified fragments.

Drought and PHS were worldly hazard in wheat growing with yield reduction and quality degradation. In our study, two genes *Dreb1* and *TaCRT-D* related to drought tolerance, and two genes *TaAFP-1B* and *Vp1B* associated with PHS tolerance were used to evaluate the stress tolerance of the 23 wheat cultivars/lines (Table S3; **Table 5**). The marker *DF/DR* for the transcription factor *TaCRT-D* and five markers *P18*, *P20*, *P21*, *P22* and *P25* for the gene *Dreb1* amplify target bands in all tested wheat accessions, indicating that these 23 wheat cultivars/lines might have the drought resistance to the extent (**Table 5**, **Figure 4**). Moreover, the marker *Vp1B3* for the PHS gene *Vp1B* could produce three types of bands, including 652 bp (PHS-susceptible types *Vp1B-a*), 845 bp (PHS-resistant types *Vp1B-b*) and 569 bp (PHS-resistant types *Vp1B-c*) (Yang et al., 2007). The marker *Vp1b2* for *Vp1B* could amplify the target bands of 532 bp (PHS-susceptible types *Vp1B-a*) and 616 bp (PHS-resistant types *Vp1B-f*) (Chang et al., 2010). Then, the marker *AFP-BF* for *TaAFP-B* could produce bands of 203 bp (PHS-resistant types *TaAFP-Bb*) and 207 bp (PHS-susceptible types *TaAFP-Ba*) (Feng et al., 2019). In our study, Yannong 161, Yannong 301, Yannong 836 and Yannong 1212 carried the PHS-resistant elite alleles *Vp-1Bc/Vp-1Bf/TaAFP-1Bb*, 13 cultivars/lines carried *Vp-1Bc/Vp-1Bf* and Yannong 572 carried only *TaAFP-1Bb*. Whereas Yannong 30, Yannong 377, Yannong 390, Yannong 745 and Yannong 1766 carried the PHS-susceptible alleles *Vp-1Ba/TaAFP-1Ba* (**Table 5**, **Figure 4**).

**Table 5:** Detection of drought resistance genes and pre-harvest sprouting (PHS) resistance genes in 23 Yannong cultivars/lines.

Cultivar/line	Drought resistance gene		Pre-sprouting harvest (PHS) resistance gene					
	*TaDREB1*	*Ta-CRT*	*Vp-1Ba*	*Vp-1Bb*	*Vp-1Bc*	*Vp-1Bf*	*TaAFP-B1a*	*TaAFP-B1b*
Yannong 15	+	+	–	–	+	+	+	–
Yannong 17	+	+	–	–	+	+	+	–
Yannong 23	+	+	–	–	+	+	+	–
Yannong 24	+	+	–	–	+	+	+	–
Yannong 30	+	+	+	–	–	–	+	–
Yannong 31	+	+	–	–	+	+	+	–
Yannong 37	+	+	–	–	+	+	+	–
Yannong 161	+	+	–	–	+	+	–	+
Yannong 191	+	+	–	–	+	+	+	–
Yannong 215	+	+	–	–	+	+	+	–
Yannong 301	+	+	–	–	+	+	–	+
Yannong 377	+	+	+	–	–	–	+	–
Yannong 390	+	+	+	–	–	–	+	–
Yannong 572	+	+	+	–	–	–	–	+
Yannong 745	+	+	+	–	–	–	+	–
Yannong 836	+	+	–	–	+	+	–	+
Yannong 999	+	+	–	–	+	+	+	–
Yannong 1212	+	+	–	–	+	+	–	+
Yan 2415	+	+	–	–	+	+	+	–
Yannong 5158	+	+	–	–	+	+	+	–
Lumai 21	+	+	–	–	+	+	+	–
Lumai 14	+	+	–	–	+	+	+	–
Yannong 1766	+	+	+	–	–	–	+	–

+, gene present; –, gene absent.

## Discussion

Yannong series wheat cultivars/lines, developed by Shandong Yantai Academy of Agricultural Sciences, play a key role in Chinese wheat production. Due to the excellent breeding foundation and unique ecological conditions in Jiaodong region, Yannong series wheat cultivars/lines have the distinct characteristics of high yield, diverse types, drought resistance, disease resistance and broad adaptability. More than 20 wheat cultivars with independent intellectual property rights have been released successively, whose extending area covers the three major wheat producing regions, including the south of Huang-Huai valley winter wheat region, the north of Huang-Huai valley winter wheat region and the northern winter wheat region (Liu et al., 2019). Especially, Yannong 1212 is the first wheat cultivar in China with a yield of more than 12,000 kilograms per ha (http://ah.people.com.cn/n2/2022/0620/c358428-40002682.html). Therefore, identifying the genetic basis of Yannong series wheat cultivars/lines is helpful to make better use of them. Molecular markers detection provided novel strategies for wheat breeding in a short span of time. As for many traits, MAS is an efficient tool to select and predict phenotype with the advantages enabling the selection of plants with desirable traits precisely, without being greatly affected by the environment (Gupta et al., 2010). In the present study, 58 molecular markers for 40 genes were selected to test with 23 representative Yannong series wheat cultivars/lines to provide theoretical evidence. The results showed that most of the tested wheat cultivars/lines carried the elite alleles of dwarfing genes, vernalization genes, PHS-resistance genes, disease-resistance genes and stress tolerance genes, which can provide the molecular evidence for the characteristics of Yannong wheat cultivars/lines.

Powdery mildew and stripe rust have been prevalent in the world, resulting in serious losses of wheat every year (Kokhmetova et al., 2014; Zhang et al., 2016). Developing resistant cultivars is an effective method to control diseases and has always been a principal breeding goal of wheat. However, numerous wheat cultivars popularized in production are highly susceptible to moderately susceptible to diseases. For example, Huang et al. (2020) reported that more than half were susceptible to stripe rust *Pst* races in 66 wheat cultivars that were widely grown in the Huang-Huai-Hai region of China during 2000 to 2016 (Huang et al., 2020). Wu et al. (2021) identified the resistance of 69 main wheat cultivars in Yunnan Province to powdery mildew with six different *Bgt* isolates at the seedling stage. The results showed that only four (5.8%) of 69 cultivars were resistant to six tested *Bgt* isolates, revealing that these Yunnan wheat cultivars have poor resistance to powdery mildew (Wu et al., 2021). In our study, the resistance level of Yannong series wheat cultivars/lines to nine *Bgt* isolates was evaluated at the seedling stage. The results showed that the overall resistance level at seedling stage were mediocre. However, the alleles of some *Pm* and *Yr* genes are still detected in quite a few cultivars/breeding lines. This may be associated with slow resistance performance in most of the Yannong series wheat cultivars/lines. As a whole, coordination of resistance and yield is important. Meanwhile, to improve the resistance level of wheat cultivars, it is necessary to pyramid some other effective genes into new cultivars without affecting the other traits. In addition, in this study, the cloned *Pm2* was detected in wheat cultivars Yannong 31 and Yannong 377. Further sequencing analysis showed that their sequences were same with cloned *Pm2*. It is likely that there is only one resistant haplotype at *Pm2* locus in hexaploid wheat (Jin et al., 2021; Yu et al., 2022). Notably, eight wheat cultivars/lines, including Yannong 15, Yannong 23, Yannong 24, Yannong 301, Yannong 745, Yannong 999, Yan 2415 and Lumai 21, showed resistance to one or multiple *Bgt* isolate, yet carried no tested *Pm* genes. These wheat cultivars/lines could be further studied by genetic analysis and map-based cloning to determine the novel gene(s), which was significant to wheat resistance breeding.

PHS caused serious threat to wheat yield and quality in many wheat producing areas, especially where continuous seasonal rainfall occurs during harvest. Recently, with the climate changing and uncertain weather conditions, PHS has become more severe and frequent (Zhang Y. J. et al., 2017). Breeding PHS-resistant wheat cultivars are regarded as a major breeding goal in many countries including China, USA, Japan, Australia and Canada (Zhang Y. J. et al., 2017). The genetic background of PHS-resistance gene is complex and always affected by multiple factors, such as morphology of spike, drying characteristics, seed dormancy characteristics, GA content, seed coat characteristics and etc. (Barnard, 2001; Torada and Amano, 2002). Therefore, it is quite difficult to accurately select the PHS-resistant genotypes in breeding. Alternatively, the development of molecular marker technology provides a simple and effective means for field breeding. Feng et al. (2008) detected 258 mini-core collections of Chinese wheat varieties using the STS marker *Vp1B3* and the results showed that among the 258 genotypes tested, 89.5% wheat varieties were amplified the same fragment ‘a’ (13.9%), ‘c’ (41.1%), ‘e’ (34.5%) as previous investigation and 2.4% varieties were new polymorphic fragments which were found as ‘b’, ‘d’, ‘f’ fragment (Feng et al., 2008). Guo et al. (2009) identified the evolution of different *Vp1B* alleles in Shandong wheat accessions by analyzing 385 landraces, 101 old cultivars and 25 current wheat cultivars using the marker *Vp1B3* (Guo et al., 2009). In our study, two markers *Vp1B3* and *Vp1b2* for gene *Vp1B* and marker *AFP-BF* for gene *TaAFP-B* were used to evaluate the distribution of PHS-resistance gene in 23 Yannong wheat cultivars/lines. The results indicated, except for Yannong 30, Yannong 377, Yannong 390, Yannong 745 and Yannong 1766, the rest (78.26%) carried at least one of the elite alleles *Vp-1Bc/Vp-1Bf/TaAFP-1Bb*. Possibly, these cultivars resistant to PHS are preferred to be selected under the environment pressure of unique ecological conditions in Jiaodong region.

Therefore, Yannong series wheat cultivars/lines carry abundant elite alleles, which is the reason for its high yield and wide adaptability. Our study revealed the genetic basis of Yannong series wheat cultivars/lines using molecular markers and provided valuable information for wheat breeding programs.

## Conclusion

In the present study, we identified the genetic basis of 23 Yannong series wheat cultivars/lines using 58 molecular markers for 40 genes from self-characteristics, broad adaptability, disease resistance and stress tolerance. Our study provided reference information and theoretical basis for wheat resistance molecular breeding.

## Data availability statement

The original contributions presented in the study are included in the article/**Supplementary Material**. Further inquiries can be directed to the corresponding authors.

## Author contributions

PM, ML and NS conceived the research. LX, NS, WL and HW performed the experiments. JL, DL, JZ, DW, YY, YL and LL developed the experimental materials. LX, NS, WL and HS performed the phenotypic assessment. YJ wrote the manuscript. All authors read and approved the final manuscript. All authors contributed to the article and approved the submitted version.

## Funding

This research was financially supported by the National Natural Science Foundation of China (32072053), the Key Research and Development Project of Shandong Province (2020CXGC010805) and Key Research and Development Project of Yantai City (2022XCZX092).

## Acknowledgments

We are grateful to Prof. Caixia Lan, College of Plant Science and Technology, Huazhong Agricultural University and Prof. Hongxing Xu, School of Life Sciences, Henan University for providing the donors with the known *Yr* genes and *Blumeria graminis* f. sp. *tritici* isolates.

## Conflict of interest

The authors declare that the research was conducted in the absence of any commercial or financial relationships that could be construed as a potential conflict of interest.

## Publisher’s note

All claims expressed in this article are solely those of the authors and do not necessarily represent those of their affiliated organizations, or those of the publisher, the editors and the reviewers. Any product that may be evaluated in this article, or claim that may be made by its manufacturer, is not guaranteed or endorsed by the publisher.
