# Supplementary material for: Genetic basis analysis of key Loci in 23 Yannong series wheat cultivars/lines
Source: Front Plant Sci. 2022 Oct 10;13:1037027. doi: 10.3389/fpls.2022.1037027 (PMC9589233; doi:10.3389/fpls.2022.1037027)
Supplement: Supplementary file 1 [file DataSheet_1.docx]

**Table S1 Pedigree and released information of 23 Yannong cultivars/lines**

| **Cultivar/line** | **Pedigree** | **Released number** |
| --- | --- | --- |
| Yannong 15 | Youbaomai/(St2422//464) | Shandong (1982) |
| Yannong 17 | Zhengmai 366/Tainong 18 | Shandong Province regional trail the 2^nd^ year |
| Yannong 23 | Yan 1061/Lumai 14 | Shandong (2003) |
| Yannong 24 | Shan 229/Anmai 1 | Shandong (2004) |
| Yannong 30 | Yannong 09135/Jimai 22 | National regional trail the 2^nd^ year |
| Yannong 31 | Yannong 09135/Jimai 22 | Shandong Province production test |
| Yannong 37 | Lumai 21/Jimai 22 | Shandong Province regional trail the 2^nd^ year |
| Yannong 161 | Jimai 22/Yannong 1212 | Shandong (2021) |
| Yannong 191 | Recurrent selection populations of dwarf male | - |
| Yannong 215 | Yan 672/Yannong 999 | Shandong (2020) |
| Yannong 301 | Jimai 22/Yan 1201 | Shandong (2021) |
| Yannong 377 | By114/Yan 6089 | Shandong (2020) |
| Yannong 390 | Jimai 22/Yannong 23 | - |
| Yannong 572 | SN055849/Jimai 22 | Shandong Province production test |
| Yannong 745 | Yanong 999/Jimai 22 | National regional trail the 2^nd^ year |
| Yannong 836 | Yan 9292 selection of satellite on-board processing systems | Shandong (2010), National Authorized (2014) |
| Yannong 999 | Yanhangxuan 2/Lin 9511//Yan BLU14-15 | Shandong (2011) , South region of Huang-Huai (2016), Shanxi (2018) |
| Yannong 1212 | Yan 5072/Shi 94-5300 | Shandong (2018), Hebei (2019), Guoshenmai (2020, 2021) |
| Yan 2415 | Yan 849/Lumai 21 | Shandong (2006) |
| Yannong 5158 | Yanhang 2/Yannong 15 | Shandong (2007), Anhui (2009), Jiangsu (2010) |
| Lumai 21 | Lumai 13/Baofeng 7228 | Shandong (1996) |
| Lumai 14 | C149/F4530 | Shandong (1990), Shanxi (1992), North region of Huang-Huai (1993) |
| Yannong 1766 | Yannong 09135/Jimai 22 | National production test |

**Table S2 Wheat accessions with known powdery mildew (*Pm*) and strip rust (*Yr*) resistance gene**

| **No.** | ***Pm* gene** | **Cultivar/line** | **No.** | ***Yr* gene** | **Cultivar/line** |
| --- | --- | --- | --- | --- | --- |
| 1 | *Pm1* | Axminster/8*Cc | 1 | *Yr1* | YR1/6*AOC |
| 2 | *Pm2* | D57-5D | 2 | *Yr5* | Triticum. spelta album |
| 3 | *Pm4a* | Yuma/8*Cc | 3 | *Yr9* | Ruihua 055 |
| 4 | *Pm5e* | Xiaobaidongmai | 4 | *Yr10* | Moro |
| 5 | *Pm6* | Coker 747 | 5 | *Yr15* | Yr15/6*Avocet S |
| 6 | *Pm12* | CI14119 | 6 | *Yr17* | Yr17/6*Avocet S |
| 7 | *Pm21* | Yangmai5/Sub.6V | 7 | *Yr18* | Yr18/6*Avocet S |
| 8 | *Pm24* | Chiyacao | 8 | *Yr24* | K733 |
| 9 | *Pm33* | Am 9/3 | 9 | *Yr26* | Yangmai 5 |
| 10 | *Pm34* | Chuanmai 44 | 10 | *Yr29* | Zhongmai 527 |
| 11 | *Pm35* | Pubing 01 | 11 | *Yr30* | Opata 85 |
| 12 | *Pm42* | P63 | 12 | *Yr41* | Chuannong 19 |
| 13 | *Pm45* | D57-6D | 13 | *Yr67* | C591 |
| 14 | *Pm47* | Hongyanglazi | 14 | *YrSP* | Spaldings Prolific |
| 15 | *Pm52* | Liangxing 99 |  |  |  |

**Table S3 Information of molecular markers in this study**

| **Gene** | **Marker** | **Primer sequence (5’-3’)** | **Reference** | **Gel** |
| --- | --- | --- | --- | --- |
| **Molecular markers for powdery mildew (*Pm*) resistance genes** | | | | |
| *Pm1* | *MAG2185-F* | GCTCCACTACTTCATCATCC | (Liang et al., 2016) | PAGE |
|  | *MAG2185-R* | ACCACAACGAACACCAACCT |  |  |
| *Pm2* | *CFD81-F* | TATCCCCAATCCCCTCTTTC | (Ma et al., 2015) | PAGE |
|  | *CFD81-R* | GTCAATTGTGGCTTGTCCCT |  |  |
|  | *BWM20-F* | GCTTCATCCTCAGCTTCGTC |  | PAGE |
|  | *BWM20-R* | GGAGGAAACAAAGGCACAGA |  |  |
|  | *Pm2b-map-3-F* | ACCACAACGAACACCAACCT | (Jin et al., 2021) | PAGE |
|  | *Pm2b-map-3-R* | ACGGGTAACCATCGAGATCA |  |  |
|  | *YTU-KASP-Pm2-F* | gaaggtgaccaagttcatgctTGTTGGACGAGAAAAGGAGAAA | (Yu et al., 2022) | KASP |
|  | *YTU-KASP-Pm2-H* | gaaggtcggagtcaacggattTGTTGGACGAGAAAAGGAGAAC |  |  |
|  | *YTU-KASP-Pm2-C* | CAATTCATCTGAGGTGTTGGC |  |  |
| *Pm4a* | *Xgwm356-F* | AGCGTTCTTGGGAATTAGAGA | (Ma et al., 2004) | PAGE |
|  | *Xgwm356-R* | CCAATCAGCCTGCAACAAC |  |  |
| *Pm5e* | *WMC364-F* | ATCACAATGCTGGCCCTAAAAC | (Zhu et al., 2008) | PAGE |
|  | *WMC364-R* | CAGTGCCAAAATGTCGAAAGTC |  |  |
| *Pm6* | *CIT02g-18-F* | GGCCTTAGTGGTGATGCAGT | (Wan et al., 2020) | PAGE |
|  | *CIT02g-18-R* | GCGGCTTGTCGGTGTATAG |  |  |
|  | *CIT02g-20-F* | GCGGCTTGTCGGTGTATAG |  | PAGE |
|  | *CIT02g-20-R* | TGTTCACACAAGCAGCAAGTT |  |  |
| *Pm12/21* | *MBH1-F* | GCCATTATAGTCAAGAGTGCACTAGCTGT | (Bie et al., 2015) | PAGE |
|  | *MBH1-R* | AGCTCCTCTCGTTCTCCAATGCT |  |  |
| *Pm24* | *GWM337-F* | CCTCTTCCTCCCTCACTTAGC | (Xue et al., 2012) | PAGE |
|  | *GWM337-R* | TGCTAACTGGCCTTTGCC |  |  |
|  | *STS-Pm24-F* | TATGGTGTCATTTAAGGCTGAG | (Lu et al., 2020) | PAGE |
|  | *STS-Pm24-R* | TTTCTCACATCCTCATCAAACC |  |  |
| *Pm33* | *GWM111-F* | TCTGTAGGCTCTCTCCGACTG | (Zhu et al., 2005) | PAGE |
|  | *GWM111-R* | ACCTGATCAGATCCCACTCG |  |  |
| *Pm34* | *BARC144-F* | GCGTTTTAGGTGGACGACATAGATAGA | (Miranda et al., 2006) | PAGE |
|  | *BARC144-R* | GCGCCACGGGCATTTCTCATAC |  |  |
| *Pm35* | *CFD26-F* | TCAAGATCGTGCCAAATCAA | (Miranda et al., 2007) | PAGE |
|  | *CFD26-R* | ACTCCAAGCTGAGCACGTTT |  |  |
| *Pm42* | *Xgwm148-F* | GTGAGGCAGCAAGAGAGAAA | (Hua et al., 2009) | PAGE |
|  | *Xgwm148-R* | CAAAGCTTGACTCAGACCAAA |  |  |
| *Pm45* | *CFD80-F* | ATAGGGGTTTTGAATCACTCC | (Ma et al., 2011) | PAGE |
|  | *CFD80-R* | TTGGATTTGCAGAGCCTTCT |  |  |
| *Pm47* | *GWM46-F* | GCACGTGAATGGATTGGAC | (Xiao et al., 2013) | PAGE |
|  | *GWM46-R* | TGACCCAATAGTGGTGGTCA |  |  |
| *Pm52* | *Xicssl 326-F* | AAGATGCACTTACCCAAAAAC | (Wu et al., 2019) | PAGE |
|  | *Xicssl 326-R* | TGCTACATATAACTGCTGCTG |  |  |
|  | *Xicscl795-F* | GTCAACCTCATCTTCTCCTG |  | PAGE |
|  | *Xicscl795-R* | AGATGCATATCACATTCACG |  |  |
| **Molecular markers for stripe rust (*Yr*) resistance genes** | | | | |
| *Yr1* | *GWM372-F* | AATAGA GCCCTGGGACTGGG | (Liu et al., 2006) | Agarose gel |
|  | *GWM372-R* | GAAGGACGACATTCCACCTG |  |  |
| *Yr5* | *WMC1750-F* | GCTCAGTCAAACCGCTACTTCT | (Chen et al., 2003) | PAGE |
|  | *WMC1750-R* | CACTACTCCAATCTATCGCCGT |  |  |
| *Yr9* | *H20-F* | GTTGGAAGGGAGCTCGAGCTG | (Liu et al., 2008) | Agarose gel |
|  | *H20-R* | GTTGGGCAGAAAGGTCGACATC |  |  |
| *Yr10* | *SC200-F* | CTGCAGAGTGACATCATACA | (Shao et al., 2001) | PAGE |
|  | *SC200-R* | TCGAACTAGTAGATGCTGGC |  |  |
| *Yr15* | *Y15K1-F2* | GGAGATAGAGCACATTACAGAC | (Klymiuk et al., 2018) | Agarose gel |
|  | *UHW301R* | TTTCGCATCCCACCCTACTG |  |  |
|  | *Barc8-F* | GCGGGAATCATGCATAGGAAAACAGAA | (Peng et al., 2000) | PAGE |
|  | *Barc8-R* | GCGGGGGCGAAACATACACATAAAAACA |  |  |
| *Yr17* | *SC2372-F* | AGGGGCTACTGACCAAGGCT | (Jia et al., 2010) | Agarose gel |
|  | *SC2372-R* | TGCAGCTACAGCAGTATGTACACAAAA |  |  |
| *Yr18* | *csLV34-F* | GTTGGTTAAGACTGGTGATGG | (Lagudah et al., 2006) | Agarose gel |
|  | *csLV34-R* | TGCTTGCTATTGCTGAATAGT |  |  |
| *Yr24* | *Xgwm11-F* | GGATAGTCAGACAATTCTTGTG | (Liu et al., 2005) | PAGE |
|  | *Xgwm11-R* | GTGAATTGTGTCTTGTATGCTTCC |  |  |
| *Yr26* | *WE173-F* | GGGACAAGGGGAGTTGAAGC | (Wang et al., 2008) | PAGE |
|  | *WE173-R* | GAGAGTTCCAAGCAGAACAC |  |  |
| *Yr29* | *csLV46-F* | CGAGACGTCGTCTTCTCTAAC | (Ren et al., 2017) | PAGE |
|  | *csLV46-R* | GTGTATGTGTTGATTCTCCTCG |  |  |
| *Yr30* | *Xgwm533-F* | GTTGCTTTAGGGGAAAAGCC | (Hayden et al., 2004) | Agarose gel |
|  | *Xgwm533-R* | AAGGCGAATCAAACGGAATA |  |  |
| *Yr41* | *Xgwm410-F* | GCTTGAGACCGGCACAGT | (Luo et al., 2008) | PAGE |
|  | *Xgwm410-R* | CGAGACCTTGAGGGTCTAGA |  |  |
|  | *Xgwm374-F* | ATAGTGTGTTGCATGCTGTGTG |  | PAGE |
|  | *Xgwm374-R* | TCTAATTAGCGTTGGCTGCC |  |  |
| *Yr67* | *Xbarc182-F* | CCATGGCCAACAGCTCAAGGTCTC | (Xu et al., 2014) | PAGE |
|  | *Xbarc182-R* | CGCAAAACCGCATCAGGGAAGCACCAAT |  |  |
|  | *Xcfa2040-F* | TCAAATGATTTCAGGTAACCACTA |  | PAGE |
|  | *Xcfa2040-R* | TTCCTGATCCCACCAAACAT |  |  |
| *YrSP* | *dp269-F* | CTGCTGTCACCGCTCTCC | (Feng et al., 2015) | PAGE |
|  | *dp269-R* | AGTCACACGCCCTACTCTCC |  |  |
| **Molecular markers for pre-sprouting harvest(PHS) resistance genes** | | | | |
| *Vp-1B* | *Vp1B3-F* | TGCTCCTTTCCCAATTGG | (Yang et al., 2007) | PAGE |
|  | *Vp1B3-R* | ACCCTCCTGCAGCTCATTG |  |  |
|  | *Vp1-b2-F* | TGCTCCTTTCCCAATTGG | (Chang et al., 2010) | PAGE |
|  | *Vp1-b2-R* | TGCTTCTCTTCTCTCACCAGTG |  |  |
| *TaAFP-B* | *AFPB-F* | CTTCCTGAGAATTTGGCCGT | (Feng et al., 2019) | PAGE |
|  | *AFPB-R* | TGAGCTCGACCACCTCGTCG |  |  |
| **Molecular markers for drought resistance genes** | | | | |
| *Dreb1* | *P18-F* | CCCAACCCAAGTGATAATAATCT | (Wei. 2007) | PAGE |
|  | *P18-R* | TTGTGCTCCTCATGGGTACTT |  |  |
|  | *P20-F* | TCGTCCCTCTTCTCGCTCCAT |  |  |
|  | *P20-R* | GCGGTTGCCCCATTAGACATAG |  |  |
|  | *P21-F* | CGGAACCACTCCCTCCATCTC |  |  |
|  | *P21-R* | CGGTTGCCCCATTAGACGTAA |  |  |
|  | *P22-F* | CTGGCACCTCCATTGCCGCT |  |  |
|  | *P22-R* | AGTACATGAACTCAACGCACAGGACAAC |  |  |
|  | *P25-F* | CTGGCACCTCCATTGCTGCC |  |  |
|  | *P25-R* | AGTACATGAACTCAACGCACAGGACAAC |  |  |
| *TaCRT-D* | *DF* | GTGGGACTCAAACAAAGAAG | (Wang et al., 2017) | PAGE |
|  | *DR* | TTAGAACTGAATGATGCATT |  |  |
| **Molecular markers for dwarfing genes** | | | | |
| *Rht-B1b* | *BF* | GGTAGGGAGGCGAGAGGCGAG | (Ellis et al., 2002) | PAGE |
|  | *MR1* | CATCCCCATGGCCATCTCGAGCTA |  |  |
| *Rht-D1b* | *DF* | CGCGCAATTATTGGCCAGAGATAG |  |  |
|  | *MR2* | CCCCATGGCCATCTCGAGCTGCTA |  |  |
| *Rht8* | *GWM-261-F* | CTCCCCTGTACGCCTAAGGC | (Korzun et al., 1998) | PAGE |
|  | *GWM-261-R* | CTCGCGCTACTAGCCATTG |  |  |
| **Molecular markers for vernalization genes** | | | | |
| *Vrn-A1c* | *Intr1/A/F2* | AGCCTCCACGGTTTGAAAGTAA | (Fu et al., 2005) | PAGE |
|  | *Intr/A/R3* | AAGTAAGACAACACGAATGTGAGA |  |  |
| *vrn-A1* | *VRN1AF* | GAAAGGAAAAATTCTGCTCG | (Yan et al., 2004) | PAGE |
|  | *VRN-INT1R* | GCAGGAAATCGAAATCGAAG |  |  |
|  | *Intr1/C/F* | GCACTCCTAACCCACTAACC | (Fu et al., 2005) | PAGE |
|  | *Intr1/AB/R* | TCATCCATCATCAAGGCAAA |  |  |
| *Vrn-B1* | *Intr/B/F* | CAAGTGGAACGGTTAGGACA |  |  |
|  | *Intr1/B/R3* | CTCATGCCAAAAATTGAAGATGA |  |  |
| *vrn-B1* | *Intr/B/F* | CAAGTGGAACGGTTAGGACA |  |  |
|  | *Intr1/B/R4* | CAAATGAAAAGGAATGAGAGCA |  |  |
| *Vrn-D1* | *Intr/D/F* | GTTGTCTGCCTCATCAAATCC |  |  |
|  | *Intr1/D/R3* | GGTCACTGGTGGTCTGTGC |  |  |
| *vrn-D1* | *Intr/D/F* | GTTGTCTGCCTCATCAAATCC |  |  |
|  | *Intr1/D/R4* | AAATGAAAAGGAACGAGAGCG |  |  |
| *Vrn-B3* | *FT-B-INS-F* | CATAATGCCAAGCCGGTGAGTAC | (Yan et al., 2006) | PAGE |
|  | *FT-B-INS-R* | ATGTCTGCCAATTAGCTAGC |  |  |
| *vrn-B3* | *FT-B-NOINS-F* | ATGCTTTCGCTTGCCATCC |  |  |
|  | *FT-B-NOINS-R* | CTATCCCTACCGGCCATTAG |  |  |

**REFERENCES**

Bie, T. D., Zhao, R. H., Zhu, S. Y., Chen, S. L., Cen, B., Zhang, B., et al. (2015). Development and characterization of an efficient breeding-practical marker *MBH1* simultaneously tagging *Pm21* and *PmV* genes conferring resistance to wheat powdery mildew. *Mol. Breed.* 35, 10-1007. doi: 10.1007/s11032-015-0385-3

Chang, C., Feng, J. M., Si, H. Q., Yin, B., Zhang, H. P., and Ma, C. X. (2010). Validating a novel allele of *viviparous-1* (*Vp-1Bf*) associated with high seed dormancy of Chinese wheat landrace, Wanxianbaimaizi. *Mol. Breed.* 25, 517-525. doi: 10.1007/s11032-009-9350-3

Chen, X. M., Soria, M. A., Yan, G. P., Sun, J., and Dubcovsky, J. (2003). Development of sequence tagged site and cleaved amplified polymorphic sequence markers for wwheat stripe rust resistance gene *Yr5*. *Crop Sci.* 43, 2058-2064. doi: 10.2135/cropsci2003.2058

Ellis, M., Spielmeyer, W., Gale, K., Rebetzke, G., and Richards, R. (2002). "Perfect" markers for the *Rht-B1b* and *Rht-D1b* dwarfing genes in wheat. *Theor. Appl. Genet.* 105, 1038-1042. doi: 10.1007/s00122-002-1048-4

Feng, J. Y., Wang, M. N., Chen, X. M., See, D. R., Zheng, Y. L., Chao, S. M., et al. (2015). Molecular mapping of *YrSP* and its relationship with other genes for stripe rust resistance in wheat chromosome 2BL. *Phytopathology* 105, 1206-1213. doi: 10.1094/phyto-03-15-0060-r

Feng, Y. M., Liu, M., Wang, Z., Zhao, X. L., Han, B., Xing, Y. P., et al. (2019). A 4-bp deletion in the 5’UTR of *TaAFP-B* is associated with seed dormancy in common wheat (*Triticum aestivum* L.). *BMC Plant Biol.* 19, 349. doi: 10.1186/s12870-019-1950-4

Fu, D. L., Szűcs, P., Yan, L. L., Helguera, M., Skinner, J. S., Zitzewitz, J. V., et al. (2005). Large deletions within the first intron in *VRN-1* are associated with spring growth habit in barley and wheat. *Mol. Genet. Genomics* 273, 54-65. doi: 10.1007/s00438-004-1095-4

Hayden, M. J., Kuchel, H., and Chalmers, K. J. (2004). Sequence tagged microsatellites for the *Xgwm533* locus provide new diagnostic markers to select for the presence of stem rust resistance gene *Sr2* in bread wheat (*Triticum aestivum* L.). *Theor. Appl. Genet.* 109, 1641-1647. doi: 10.1007/s00122-004-1787-5

Hua, W., Liu, Z. J., Zhu, J., Xie, C. J., Yang, T., Zhou, Y. L., et al. (2009). Identification and genetic mapping of *pm42*, a new recessive wheat powdery mildew resistance gene derived from wild emmer (*Triticum turgidum* var. *dicoccoides*). *Theor. Appl. Genet.* 119, 223-230. doi: 10.1007/s00122-009-1031-4

Jia, J. Q., Lei, M. P., Liu, C., Li, G. R., and Yang, Z. J. (2010). Exploitation and application of a new SCAR marker linked to strip rust resistance gene *Yr17* in wheat. *J. Triticeae Crops* 01, 11-16. doi: 10.1080/00949651003724790

Jin, Y. L., Shi, F. Y., Liu, W. H., Fu, X. Y., Gu, T. T., Han, G. H., et al. (2021). Identification of resistant germplasm and detection of genes for resistance to powdery mildew and leaf rust from 2,978 wheat accessions. *Plant Dis.* 105, 3900-3908. doi: 10.1094/pdis-03-21-0532-re

Klymiuk, V., Yaniv, E., Huang, L., Raats, D., Fatiukha, A., Chen, S. S., et al. (2018). Cloning of the wheat *Yr15* resistance gene sheds light on the plant tandem kinase-pseudokinase family. *Nat. Commun.* 9, 3735. doi: 10.1038/s41467-018-06138-9

Korzun, V., Röder, M. S., Ganal, M. W., Worland, A. J., and Law, C. N. (1998). Genetic analysis of the dwarfing gene (*Rht8*) in wheat. Part I. Molecular mapping of *Rht8* on the short arm of chromosome 2D of bread wheat (*Triticum aestivum* L.). *Theor. Appl. Genet*. 96, 1104-1109. doi: 10.1007/s001220050845

Lagudah, E. S., McFadden, H., Singh, R. P., Huerta-Espino, J., Bariana, H. S., and Spielmeyer, W. (2006). Molecular genetic characterization of the *Lr34/Yr18* slow rusting resistance gene region in wheat. *Theor. Appl. Genet.* 114, 21-30. doi: 10.1007/s00122-006-0406-z

Liang, J. C., Fu, B. S., Tang, W. B., Khan, N. U., Li, N., and Ma, Z. Q. (2016). Fine mapping of two wheat powdery mildew resistance genes located at the *Pm1* cluster. *Plant Genome* 9, 1-9. doi: 10.3835/plantgenome2015.09.0084

Liu, C., Yang, Z. J., Li, G. R., Zeng, Z. X., Zhang, Y., Zhou, P. J., et al. (2008). Isolation of a new repetitive DNA sequence from *Secale africanum* enables targeting of *Secale* chromatin in wheat background. *Euphytica* 159, 249-258. doi: 10.1007/s10681-007-9484-5

Liu, Y. P., Cao, S. H., Wang, X. P., Xu, Z. B., Zhang, X. Q., and Jin, J. X. (2005). Molecular mapping of stripe rust resistance gene *Yr24* in wheat. *Acta Phytopathol. Sin.* 5, 478-480. doi: 10.13926/j.cnki.apps.2005.05.019

Liu, Y., Zhang, Z. Y., Xin, Z. Y., Lin, Z. S., Du, L. P., Xu, H. J., et al. (2006). Analysis on the rust resistance genes of a new wheat germplasm YW243 by molecular markers. *Sci. Agric. Sin.* 2, 295-299. doi: 10.1360/aps040178

Lu, P., Guo, L., Wang, Z. Z., Li, B. B., Li, J., Li, Y. H., et al. (2020). A rare gain of function mutation in a wheat tandem kinase confers resistance to powdery mildew. *Nat. Commun.* 11, 680. doi: 10.1038/s41467-020-14294-0

Luo, P. G., Hu, X. Y., Ren, Z. L., Zhang, H. Y., Shu, K., and Yang, Z. J. (2008). Allelic analysis of stripe rust resistance genes on wheat chromosome 2BS. *Genome* 51, 922-927. doi: 10.1139/g08-079

Ma, H. Q., Kong, Z. X., Fu, B. S., Li, N., Zhang, L. X., Jia, H. Y., et al. (2011). Identification and mapping of a new powdery mildew resistance gene on chromosome 6D of common wheat. *Theor. Appl. Genet.* 123, 1099. doi: 10.1007/s00122-011-1651-3

Ma, P. T., Xu, H. X., Xu, Y. F., Li, L. H., Qie, Y. M., Luo, Q. L., et al. (2015). Molecular mapping of a new powdery mildew resistance gene *Pm2b* in Chinese breeding line KM2939. *Theor. Appl. Genet.* 128, 613-622. doi: 10.1007/s00122-015-2457-5

Ma, Z. Q., Wei, J. B., and Cheng, S. H. (2004). PCR-based markers for the powdery mildew resistance gene *Pm4a* in wheat. *Theor. Appl. Genet.* 109, 140-145. doi: 10.1007/s00122-004-1605-0

Miranda, L. M., Murphy, J. P., Marshall, D., Cowger, C., and Leath, S. (2007). Chromosomal location of *Pm35*, a novel *Aegilops tauschii* derived powdery mildew resistance gene introgressed into common wheat (*Triticum aestivum* L.). *Theor. Appl. Genet.* 114, 1451-1456. doi: 10.1007/s00122-007-0530-4

Miranda, L. M., Murphy, J. P., Marshall, D., and Leath, S. (2006). *Pm34*: a new powdery mildew resistance gene transferred from *Aegilops tauschii* Coss. to common wheat (*Triticum aestivum* L.). *Theor. Appl. Genet.* 113, 1497-1504. doi: 10.1007/s00122-006-0397-9

Peng, J. H., Fahima, T., Röder, M. S., Huang, Q. Y., Dahan, A., Li, Y. C., et al. (2000). High-density molecular map of chromosome region harboring stripe-rust resistance genes *YrH52* and *Yr15* derived from wild emmer wheat, *Triticum dicoccoides*. *Genetica* 109, 199-210. doi: 10.1023/a:1017573726512

Ren, Y., Singh, R. P., Basnet, B. R., Lan, C. X., Huerta-Espino, J., Lagudah, E. S., et al. (2017). Identification and mapping of adult plant resistance loci to leaf rust and stripe rust in common wheat cultivar Kundan. *Plant Dis.* 101, 456-463. doi: 10.1094/pdis-06-16-0890-re

Shao, Y. T., Niu, Y. C., Zhu, L. H., Cui, W. X., and Wu, L. R. (2001). AFLP marker of wheat stripe rust resistance gene *Yr10*. *Chin. Sci. Bull.* 8, 669-672. doi: 10.3321/j.issn:0023-074x.2001.08.012

Wan, W. T., Xiao, J., Li, M. L., Tang, X., Wen, M. X., Cheruiyot, A. K., et al. (2020). Fine mapping of wheat powdery mildew resistance gene *Pm6* using 2B/2G homoeologous recombinants induced by the *ph1b* mutant. *Theor. Appl. Genet.* 133, 1265-1275. doi: 10.1007/s00122-020-03546-8

Wang, C. M., Zhang, Y. P., Han, D. J., Kang, Z. S., Li, G. P., Cao, A. Z., et al. (2008). SSR and STS markers for wheat stripe rust resistance gene *Yr26*. *Euphytica* 159, 359-366. doi: 10.1007/s10681-007-9524-1

Wang, J. P., Li, R. Z., Mao, X. G., and Jing, R. L. (2017). Functional analysis and marker development of *TaCRT-D* gene in common wheat (*Triticum aestivum* L.). *Front. Plant Sci.* 8, 1557. doi: 10.3389/fpls.2017.01557

Wei, B. (2006). SNP marker developing and mapping of *TaDREB1* gene related to drought resistance in wheat. *Northwest A & F University MA thesis*. [https/kns.cnki.net/KCMS/detail/detail.aspx?dbname=CMFD2007&filename=2006179852.nh](https://kns.cnki.net/KCMS/detail/detail.aspx?dbname=CMFD2007&filename=2006179852.nh)

Wu, P. P., Hu, J. H., Zou, J. W., Qiu, D., Qu, Y. F., Li, T., et al. (2019). Fine mapping of the wheat powdery mildew resistance gene *Pm52* using comparative genomics analysis and the Chinese Spring reference genomic sequence. *Theor. Appl. Genet.* 132, 1451-1461. doi: 10.1007/s00122-019-03291-7

Xiao, M. G., Song, F. J., Jiao, J. F., Wang, X. M., Xu, H. X., and Li, H. J. (2013). Identification of the gene *Pm47* on chromosome 7BS conferring resistance to powdery mildew in the Chinese wheat landrace Hongyanglazi. *Theor. Appl. Genet.* 126, 1397-1403. doi: 10.1007/s00122-013-2060-6

Xu, H. X., Zhang, J., Zhang, P., Qie, Y. M., Niu, Y. C., Li, H. J., et al. (2014). Development and validation of molecular markers closely linked to the wheat stripe rust resistance gene *YrC591* for marker-assisted selection. *Euphytica* 198, 317-323. doi: 10.1007/s10681-014-1108-2

Xue, F., Wang, C. Y., Li, C., Duan, X. Y., Zhou, Y. L., Zhao, N. J., et al. (2012). Molecular mapping of a powdery mildew resistance gene in common wheat landrace Baihulu and its allelism with *Pm24*. *Theor. Appl. Genet.* 125, 1425-1432. doi: 10.1007/s00122-012-1923-6

Yan, L., Fu, D., Li, C., Blechl, A., Tranquilli, G., Bonafede, M., et al. (2006). The wheat and barley vernalization gene *VRN3* is an orthologue of *FT*. *Mol. Biol. Evol.* 103, 19581-19586. doi: 10.1073/pnas.0607142103

Yan, L., Helguera, M., Kato, K., Fukuyama, S., Sherman, J., and Dubcovsky, J. (2004). Allelic variation at the *VRN-1* promoter region in polyploid wheat. *Theor. Appl. Genet*. 109, 1677-1686. doi: 10.1007/s00122-004-1796-4

Yang, Y., Zhao, X. L., Xia, L. Q., Chen, X. M., Xia, X. C., Yu, Z., et al. (2007). Development and validation of a *Viviparous-1* STS marker for pre-harvest sprouting tolerance in Chinese wheats. *Theor. Appl. Genet.* 115, 971-980. doi: 10.1007/s00122-007-0624-z

Yu, Z. Y., Xiao, L. N., Su, F. Y., Liu, W., Luo, F., Han, R., et al. (2022) Mining of wheat *Pm2* alleles for goal-oriented marker-assisted breeding. *Front. Plant Sci.* 13, 912589. doi: 10.3389/fpls.2022.912589

Zhu, Y. L., Wang, L. M., and Wang, H. G. (2008). Studies on SSR molecular marker of wheat powdery mildew resistance gene *Pm5e*. *Mol. Plant Breed.* 6, 1080-1084. doi: 10.1145/1344411.1344416

Zhu, Z. D., Zhou, R. H., Kong, X. Y., Dong, Y. C., and Jia, J. Z. (2005). Microsatellite markers linked to 2 powdery mildew resistance genes introgressed from *Triticum carthlicum* accession PS5 into common wheat. *Genome* 48, 585-590. doi: 10.1139/g05-016
